# Disruption of *YPS1* and *PEP4* genes reduces proteolytic degradation of secreted HSA/PTH in *Pichia pastoris* GS115

**DOI:** 10.1007/s10295-013-1264-8

**Published:** 2013-03-26

**Authors:** Min Wu, Qi Shen, Yong Yang, Sheng Zhang, Wen Qu, Jing Chen, Hongying Sun, Shuqing Chen

**Affiliations:** 1 Institute of Pharmacology and Toxicology and Biochemical Pharmaceutics, College of Pharmaceutical Sciences, Zhejiang University, 866 Yuhangtang Road, Hangzhou, 310058 Zhejiang People’s Republic of China; 2 Robert W. Holley Center for Agriculture and Health, USDA-ARS, Ithaca, NY 14853 USA; 3 Institute of Biotechnology and Life Sciences Biotechnologies, Cornell University, Ithaca, NY 14853 USA; 4 School of Pharmaceutical Sciences, University of Shizuoka, Shizuoka, 4228526 Japan

**Keywords:** Heterologous protein expression, Proteolytic degradation, *Pichia pastoris*, Yapsin, Proteinase A

## Abstract

Human serum albumin (HSA) and human parathyroid hormone (1-34) [PTH (1-34)] fusion protein [HSA/PTH (1-34)] is a promising long-acting form of PTH (1-34) for osteoporosis treatment. Secretory expression of intact HSA/PTH (1-34) in *Pichia pastoris* GS115 was accompanied by two degradation fragments, with molecular weights around 66 kDa, in addition to the well-known ~45 kDa HSA-truncated fragment, resulting in a low yield of intact protein. In this study, two internal cleavage sites were identified in the PTH (1-34) portion of the fusion protein by Western Blot analysis. To minimize proteolytic cleavages, several protease genes including *PEP4* (encoding proteinase A), *PRB1* (proteinase B) and seven *YPSs* genes (yapsin family members) were knocked out respectively by disruption of the individual genes and the selective combinations. Reduced degradation was observed by single disruption of either *PEP4* gene or *YPS1* gene, and the lowest level of degradation was observed in a *pep4*△*yps1*△ double disruptant. After 72 h of induction, more than 80 % of the HSA/PTH (1-34) secreted by the *pep4*△*yps1*△ double disruptant remained intact, in comparison to only 30 % with the wild-type strain.

**Electronic supplementary material:**

The online version of this article (doi:10.1007/s10295-013-1264-8) contains supplementary material, which is available to authorized users.

## Introduction

Human parathyroid hormone (1-34) [PTH (1-34)] covers most of the hormonal actions of intact human parathyroid hormone [PTH (1-84)], namely regulating calcium/phosphate homeostasis and controlling bone turnover in vertebrates by activating specific receptors located on osteoblastic and renal tubular cell [16]. PTH (1-34) has now been developed as a promising agent in the treatment of osteoporosis [17, 25]. However, the short half-life of unmodified PTH (1-34) at ~1 h in humans makes frequent injection (once daily) necessary in long-term dosing regimens (1–2 years), and therefore limits its clinical applications.

Albumin fusion technology is a strategy for decreasing clearance of short-acting drugs such as PTH (1-34), providing the advantageous pharmacokinetic properties of human serum albumin (HSA) due to its long biological half-life (*t*_1/2_ of 19 days) to its fusion partner [22]. Previously, in our efforts to extend the biological half-life of native PTH (1-34), we have constructed an HSA/PTH (1-34) fusion protein via fusion of N-terminus of PTH (1-34) to C-terminus of HSA, and could obtain the recombinant biologically active fusion protein using *Pichia pastoris* expression system [4]. Unfortunately, when HSA/PTH (1-34) was expressed in *Pichia pastoris* strain GS115, two degradation fragments of around 66 kDa were found, in addition to a ~45 kDa HSA-truncated fragment. The formation of a ~45 kDa fragment is well-known from secreted production of HSA alone, in both *Saccharomyces cerevisiae* [13] and *P. pastoris* [14]. The inhomogeneous expression of HSA/PTH (1-34) fusion protein made it more difficult and more time-consuming for downstream purification of intact recombinant protein with high purity.

It has become increasingly clear that proteolytic degradation of the recombinant gene products by host-specific proteases is one of the major problems hindering effective production and purification of heterologous proteins from yeasts [21, 27]. And genetic manipulation of host strain by systematic disruption analysis of the key proteases seems to be an effective solution to decrease proteolytic degradation [5, 6, 9, 10, 15] .

Yapsins are a family of glycosylphosphatidylinositol (GPI)-linked aspartyl proteases having specificity to cleave at the C-terminal side of basic amino acids [2]. A number of studies have reported the involvement of yapsins, particularly yapsin 1, in the cleavage of various heterologous proteins produced in yeast [1, 12, 13]. It was reported that the ~45 kDa HSA-truncated fragment produced in *S. cerevisiae* [13] was attributable to yapsin 1, and a partial reduction of the similar ~45 kDa HSA-truncated fragment in *P. pastoris* [26] was also found by *YPS1* disruption. PTH (1-84) was also susceptible to yapsins when expressed in *S. cerevisiae* and the use of multiple-yapsin-deficient mutant was efficient in preventing the proteolytic degradation [5]. *YPS1* gene of *P. pastoris* was first cloned and characterised by Werten and Wolf [24], and the authors found that the *yps1*-disrupted *P. pastoris* strain was beneficial for secreting production of collagen-inspired gel-forming polymers [20]. Besides, Yao et al. [26] found that the significant reduction of HSA-AK15 (R13 K) degradation, which occurred in the sequence of AK15 (R13 K), was achieved by the *YPS1* disruption in *P. pastoris* strain.

Given this background, we hypothesized that degradation of HSA/PTH (1-34) in *P. pastoris* may be due to the yapsin family. The 9.43 Mbp genomic sequence of *P. pastoris* strain GS115 in 2009 revealed the presence of other six putative yapsin genes (*YPS2*,* YPS3*, *YPS7*, *MKC7*, *YPS* ^′^, *YPS* ^″^) [7]. Five of these were disrupted and their effects on cell-wall integrity were investigated [8]. To the best of our knowledge, the present work is the first to study the effect of the individual disruption of seven putative yapsins on the degradation of recombinant proteins in *P. pastoris*. In this study, firstly, the inhomogeneous products of HSA/PTH (1-34) were characterized by biochemical and Western blot analyses. Secondly, in order to investigate the proteolytic effect of each yapsin member, seven single-yapsin-deficient GS115 mutants were constructed. Two prominent vacuolar proteases (proteinase A and proteinase B) were also evaluated, as vacuolar proteases are often responsible for a large fraction of total cellular proteolysis [21]. As a result, we have successfully identified that both *PEP4* disruption and *YPS1* disruption were beneficial for degradation reduction of HSA/PTH (1-34) fusion protein via visualized PAGE analysis. Thirdly, to achieve efficient production of intact HSA/PTH (1-34), we constructed a double gene disruptant (proteinase A and yapsin 1 double disrupted) as an effective host strain. The double disruptant was advantageous over the wild-type strain both in shake-flask and in bioreactor fermentation, which would allow high yield of this interesting protein and thereby simplify purification processes in industrial applications.

## Materials and methods

### Strains and media

The *P. pastoris* strains used in this study are listed in Table 1. Strains were cultured in the following media: YPD (1 % yeast extract, 2 % peptone, 2 % glucose) for subcultivation; BMGY (1 % yeast extract, 2 % peptone, 1.34 % YNB, 4 × 10^−5^ % biotin, 1 % glycerol, 100 mM potassium phosphate pH 6.0) and BMMY (same as BMGY substituting 1 % glycerol with 1 % methanol) for recombinant protein production. YPD Zeocin^+^ plates (YPD plus 2 % agar and 50 μg/mL Zeocin) were used for screening of *YPS*-deficient strains. Yeast competent cells transformed with HSA/PTH (1-34) expression vector were plated onto RDB His^−^ plates (1 M sorbitol, 2 % glucose, 1.34 % YNB, 4 × 10^−5^ % biotin, 0.005 % amino acids, 2 % agar).

**Table 1:** *Pichia pastoris* strains used in this study

Strain name	Disrupted genes	Genbank accession no.	Protease family	Genotype	Reference
WT GS115	–	–	–	*his4*	Purchased from Invitrogen
GS115 *yps1*△	*YPS1*	XM_ 002493977	Aspartic-type endopeptidase	*his4 yps1*∷*Sh ble*	This study
GS115 *yps2*△	*YPS2*	XM_002492474	Aspartic-type endopeptidase	*his4 yps2*∷*Sh ble*	This study
GS115 *yps3*△	*YPS3*	XM_002492478	Aspartic-type endopeptidase	*his4 yps3*∷*Sh ble*	This study
GS115 *yps7*△	*YPS7*	XM_002942575	Aspartic-type endopeptidase	*his4 yps7*∷*Sh ble*	This study
GS115 *mkc7*△	*MKC7*	XM_002489993	Aspartic-type endopeptidase	*His4 mkc7*∷*Sh ble*	This study
GS115 *yps*′△	*YPS*′^a^	XM_002492327	Aspartic-type endopeptidase	*his4 yps*′∷*Sh ble*	This study
GS115 *yps*″△	*YPS* ^″^^a^	XM_002493054	Aspartic-type endopeptidase	*his4 yps*″∷*Sh ble*	This study
GS115 *pep4*△	*PEP4*	XM_002493288	Aspartic-type endopeptidase	*his4 pep4*∷*URA3*	Purchased from Invitrogen
GS115 *pep4prb1*△	*PEP4, PRB1*	XM_002493288, XM_002489831	Aspartic-type endopeptidase, serine-type endopeptidase	*his4 pep4*∷*URA3 prb1*∷*URA3*	Purchased from Invitrogen
GS115 *pep4*△*yps1*△	*PEP4, YPS1*	XM_002493288, XM_002493977	Aspartic-type endopeptidase, aspartic-type endopeptidase	*his4 pep4*∷*URA3 yps1*∷*Sh ble*	This study

^a^ *YPS′* and *YPS″* represent additional putative yapsin-like genes in the *P. pastoris* genome, not unequivocally attributable to previously reported yapsin homologs

### Construction of HSA/PTH (1-34) expression vector

For creating the HSA/PTH (1-34) fusion protein [Genbank accession no. **JN711437**], the C-terminus of HSA and the N-terminus of PTH (1-34) were genetically linked by a flexible linker GlyGlyGlyGlySer, as previously reported [4]. In this study, to secrete the fusion protein with its native N-terminus, the *KEX2* cleavage site was positioned precisely in front of the first aa of HSA/PTH (1-34) protein sequence. pPIC9 was chosen as the expression vector. The detailed method for plasmid construction is described in supplementary materials and methods.

### Construction of protease-deficient strains

**Fig. 1:**
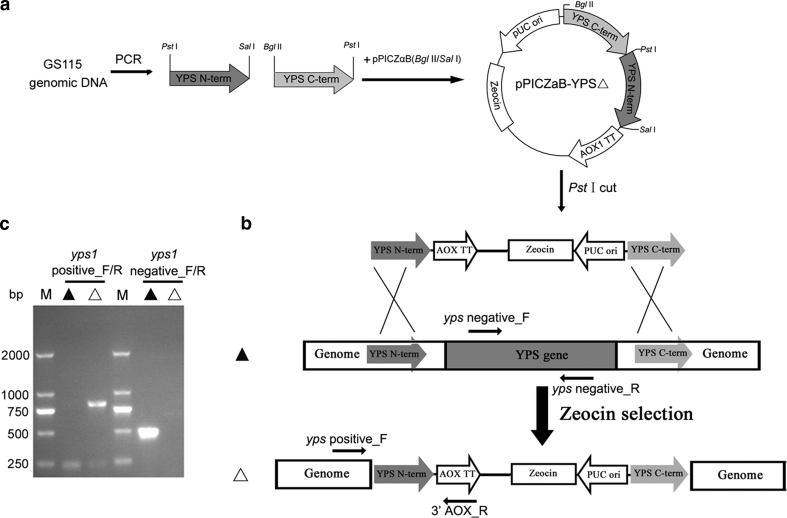
Schematic representation of experimental procedures for *YPS* gene disruption and confirmation. **a** Construction strategy for *YPS* gene disruption cassette. **b** Disruption of *YPS* gene by homologous recombination. The symbols *filled triangle* and *open triangle* indicate parental strain and the *yps* disruptant, respectively. Primer pair *yps* positive_F/3′AOX_R was used to confirm the integration of the gene disruption cassette and primer pair *yps* negative_F/*yps* negative_R was used to confirm the deletion of *YPS* gene. **c** PCR results of correct *yps1*△ transformant as an example

GS115 *pep4*△ and GS115 *pep4*△*prb1*△, also called SMD1168 and SMD1163, are commercially available (from Invitrogen, see Table 1). The seven single-yapsin-deficient GS115 mutants (GS115 *yps1*△, GS115 *yps2*△, GS115 *yps3*△, GS115 *yps7*△, GS115 *mkc7*△, GS115* yps* ^′^△, GS115* yps* ^″^△) were constructed by deleting the full ORF sequence of each yapsin gene, as shown in Fig. 1. The construction of GS115 *pep4*△*yps1*△ mutant was carried out in a similar manner from GS115 *pep4*△. Briefly, for each *YPS* gene, a 200–300 bp DNA fragment containing the 5′ homology arm of the *YPS* gene was amplified from GS115 genomic DNA using primers *YPS*_NF and *YPS*_NR. Another 200–300 bp DNA fragment containing the 3′ homology arm of the *YPS* gene was amplified using primers *YPS*_CF and *YPS*_CR. The two homology arms above were identified via DNA sequencing and sequentially subcloned into a ~2.2 kb *Bgl* II/*Sal* I plasmid fragment from pPICZαB, containing a *Sh ble* gene (zeocin resistance cassette), to give the new plasmid pPICZαB–*YPS*△. The resultant vector was then linearized with *Pst* I and introduced into competent cells of GS115 by electroporation to stimulating the homologous recombination at the corresponding *YPS* locus of the *P. pastoris* genome. Transformed cells were poured on YPD Zeocin^+^ plates and incubated at 30 °C for 3–4 days. PCR analysis was used to screen Zeocin^+^ transformants. The correct *yps* disruptant would give a specific PCR fragment with primer pair *yps* positive_F/3′AOX_R and show no specific fragment with primer pair *yps* negative_F/*yps* negative_R. All primers used for construction of pPICZαB-*YPS*△ and detection of *yps*-deficient strain are listed in Table S1.

### Transformation and screening of fusion protein expressing strains in shake flask

All *P. pastoris* strains were transformed by electroporation with *Sal* I-cut fragment of pPIC9-HSA/PTH (1-34), and plated on RDB His^−^ plates. For each strain, 3–6 clones were randomly selected and verified for integration of HSA/PTH (1-34) fusion gene by PCR. Three batches of expression were done. For each batch, seeds of all strains were grown at 30 °C to an comparative OD_600_ (OD_600_ = 2–4 for batch 1, OD_600_ = 3–4 for batch 2, OD_600_ = 2–3 for batch 3) in 20 ml BMGY before methanol induction. The cells were harvested by centrifugation at 1,500 g for 10 min, resuspended in 20 ml BMMY medium and incubated at 30 °C with constant shaking. Methanol (100 %) was added to a final concentration of 1 % every 24 h to maintain induction up to 96 h. The culture supernatant was analyzed by reducing SDS- and native PAGE.

### HSA/PTH (1-34) expression by fed-batch fermentation

For pre-culture, 50 ml YPD were inoculated with 0.5 ml frozen glycerol stock of GS115 *pep4*△*yps1*△ (or wild-type GS115) and incubated 24 h at 30 °C and 150 rpm. This first pre-culture was inoculated into 600 ml BMGY for 20–24 h at 30 °C and 150 rpm and grown to OD_600_ = 2–6. This second pre-culture was used as inoculum.

The fermentation was carried out in a 30L bioreactor (GUJS-10-30C, Orient Bioengineering Equipment and Technology Co. Ltd, China) with a working volume of 12L BSM (26.7 ml/L phosphoric acid, 0.93 g/L calcium sulfate, 18.2 g/L potassium sulfate, 14.9 g/L magnesium sulfate–7H_2_O, 4.13 g/L potassium hydroxide, 40 g/L glycerol) supplemented with 4.35 ml/L PTM_1_ trace salts (6.0 g/L cupric sulfate–5H_2_O, 0.08 g/L sodium iodide, 3.0 g/L manganese sulfate–H_2_O, 0.2 g/L sodium molybdate–2H_2_O, 0.02 g/L boric acid, 0.5 g cobalt chloride, 20.0 g/L zinc chloride, 65.0 g/L ferrous sulfate–7H_2_O, 0.2 g/L biotin, 5.0 ml sulfuric acid). The temperature was maintained at 28 °C and the pH at 5.5 (controlled with ammonium hydroxide). The dissolved oxygen (DO) was set above 20 %. A 6-hour glycerol fed-batch was performed at a feed rate of 217.8 ml glycerol/h (containing 12 ml/L PTM_1_ trace salts) to increase cell mass. After the glycerol fed-batch, cells were hungered for 2 h to fully adapt to methanol, which was confirmed by a sharp decrease in DO. During the methanol fed-batch, methanol supplemented with 12 ml/L PTM_1_ and 2 % casamino acids was used and the final concentration of methanol was maintained at 0.5 % by controlling the feeding rate.

### Gel electrophoresis (PAGE) and western blot analysis

Culture supernatant was obtained after centrifuging 1 ml of culture broth. For reducing SDS-PAGE analysis, the culture supernatant was mixed with an appropriate volume of 5 × reducing-PAGE loading buffer containing 60 mM Tris–HCl (pH 6.8), 0.1 % (W/V) bromophenol blue, 25 % (V/V) glycerol, 2 % (W/V) SDS and 14.4 mM β-mercaptoethanol. The mixed samples were then boiled for 5 min and loaded on a 8 % SDS/polyacrylamide gel. For native PAGE analysis, the culture supernatant was mixed with an appropriate volume of 5 × native-PAGE loading buffer containing 312.5 mM Tris–HCl (pH 6.8), 0.1 % (W/V) bromophenol blue and 25 % (V/V) glycerol. The mixed samples were directly loaded on a 8 % polyacrylamide gel. The gels were stained with Coomassie brilliant Blue R-250.

For Western blotting, proteins separated by native PAGE gel were electrophoretically transferred to a PVDF membrane. Primary antibodies were rabbit anti-PTH (1-17) polyclonal antibody, guinea pig anti-PTH (17-34) polyclonal antibody and rabbit anti-HSA polyclonal antibody (seek Supplementary Materials and Methods for the generation of the above three antibodies.) in three separate experiments. Secondary antibodies were HRP conjugated IgG from goat against rabbit and guinea pig, respectively. Immunoreactions were detected using 3,3′-diaminobenzidine (DAB).

### Protein relative quantization in PAGE gel

The proportion of intact (*i* fragment)and degraded HSA/PTH (1-34) (*d1* fragment and *d2* fragment) in the total protein was determined by scanning and analyzing the Coomassie blue-stained PAGE gel with laser densitometry (Bio-Rad Universal Hood II scanner) and Quantity One 1-D Analysis Software version 4.5.0 (Bio-Rad). The band densities were calculated by trace-tracking and Gauss-modeling calibration according to manufacturer’s instruction.

### Molecular weight assay of intact HSA/PTH (1-34)

The *i* fragment was obtained by a two-step chromatography purification (Phenyl Sepharose FF, Q Sepharose XL, GE Healthcare) for MALDI-TOF mass spectrometry. Native PAGE analysis of the final purified sample is provided as Fig. S3. Sample was mixed with Sinapic Acid (10 μg/ul dissolved in 30 % acetonitrile and 0.3 % TFA), spotted on a target plate and analyzed on a 5800 MALDI-TOF/TOF analyzer (AB Sciex). Linear mode with 20 kV acceleration voltage was used, with BSA as internal calibration. For the MS spectrum, 1,000 laser shots were accumulated.

## Results

### PAGE analysis of secreted HSA/PTH (1-34) from GS115

**Fig. 2:**
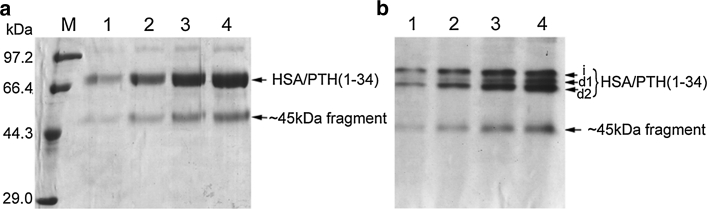
Time-course analysis of secreted HSA/PTH (1-34) in GS115. **a** reducing SDS-PAGE.**b** native PAGE. *Lane M* molecular weight marker; *lane1–4* culture supernatants after 24, 48, 72, 96 h methanol induction

**Fig. 3:**
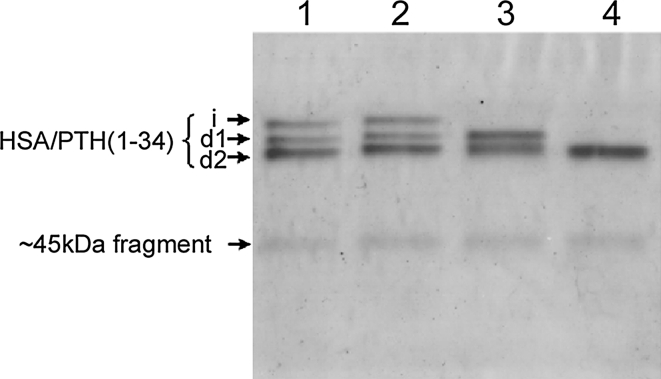
Stability of HSA/PTH (1-34) inductive supernatant incubated at 37 °C for different times by native PAGE analysis. *Lane 1* 37 °C 0 h; *lane 2* 60 °C 1 h prior to 37 °C 24 h; *lane 3* 37 °C 24 h; *lane 4* 37 °C 48 h

The GS115 strain harboring one copy of HSA/PTH (1-34) gene was methanol-induced for 96 h by ordinary shake-flask. The culture supernatants at different induction times were loaded on reducing SDS-PAGE or native PAGE. Figure 2 shows that in addition to ~45 kDa HSA-truncated fragment, there were three bands on native PAGE (Fig. 2b, band *i*, *d1*, *d2*) and they appeared as a broad band around 66 kDa on reducing SDS-PAGE (Fig. 2a). To test the stability of expressed HSA/PTH (1-34) in the GS115 supernatants, the 3-day induced supernatant was subsequently filtrated using a 0.22 μm filter membrane. The cell-free supernatant was then incubated for up to 48 h at 37 °C. Bands *i* and *d1* disappeared gradually after 48 h incubation and were eventually converted to band *d2* (Fig. 3, lane 4). In addition, the incubation time course (24 h vs. 48 h) showed that band *i* was less stable than *d1*. But when the supernatant was incubated at 60 °C for 1 h prior to 37 °C incubation, bands *i* and *d1* appeared to be relatively stable (Fig. 3, lane 2). Since pre-incubation of the supernatant at higher temperature such as 60 °C helps to reduce protease activities in the culture supernatant [3], our observations suggest that the heterogeneity of HSA/PTH (1-34) fusion protein during induction is probably caused by proteolytic degradation.

### Western blot analysis

Before Western blot analysis, N-terminal sequence of fragments *i* and *d2* were performed. Both of them showed identical sequence of DAHKS…, which matches exactly the N-terminus of mature HSA, indicating correct N-terminal process.

**Fig. 4:**
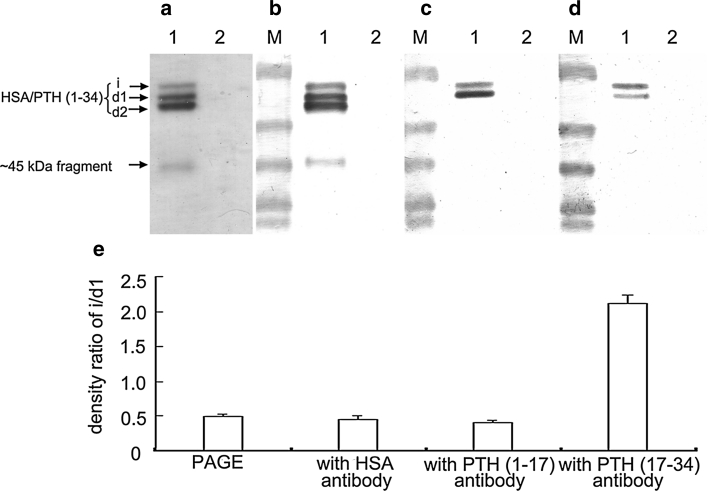
Western blot analysis of HSA/PTH (1-34) products. **a** native PAGE analysis; **b** immunoreaction with HSA antibody; **c** immunoreaction with PTH (1-17) antibody; **d** immunoreaction with PTH (17-34) antibody. *Lane M* prestained molecular weight marker; *lane 1* inductive supernatant from strain GS115 transformed with pPIC9-HSA/PTH (1-34); *lane 2* inductive supernatant from strain GS115 transformed with pPIC9 as a negative control. **e** Relative band density ratio of *i*/*d1*. Band densities are quantified by densitometric analyses with Quantity One Software version 4.5.0 (Bio-Rad). Data are mean ± S.D from three independent experiments

**Fig. 5:**
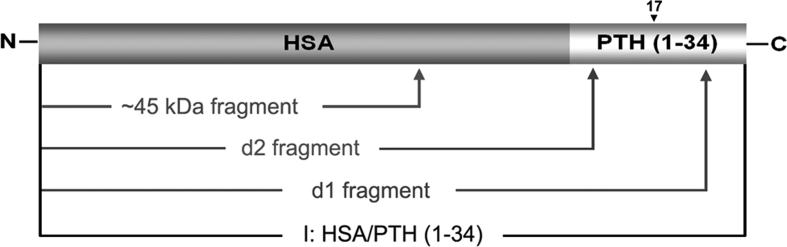
Schematic representation of cleavage sites in HSA/PTH (1-34). Number 17 refers to the aa positions in the PTH (1-34) sequence

Two PTH antibodies [anti-PTH (1-17) and anti-PTH (17-34)] were utilized for further identification of the integrity of PTH (1-34) portion of fusion protein and its degraded products. As expected, Western blot analysis showed that all the three bands (*i*, *d1* and *d2*) reacted equally well with HSA antibody (Fig. 4a, b). However, as shown in Fig. 4c, d, band *d2* reacted neither with PTH (1-17) antibody nor with PTH (17-34) antibody. Band *i* reacted with both antibodies of PTH as did band *d1*. Moreover, band *d1* showed equal affinity to PTH (1-17) antibody, but lower affinity to the PTH (17-34) as compared to band *i* (Fig 4b, c ,d). The band density ratios of *i*/*d1* in the immunoreaction with PTH (1-17) antibody and HSA antibody were both around 0.4, whereas in the immunoreaction with PTH (17-34) antibody, the density ratio of *i*/*d1* was about 2, as plotted in Fig. 4e. From the combined results above we speculate that band *i* consists of intact HSA/PTH (1-34), while band *d1* and *d2* are degradation fragments from the cleavage at the C-terminal region of fusion protein. The cleavage sites of HSA/PTH (1-34) resulting in degradation fragments *d1* and *d2* are supposedly within the PTH (17-34) and the PTH (1-17) portions, respectively, as illustrated in Fig. 5.

### Mass spectrometry of intact HSA/PTH (1-34) fusion protein

**Fig. 6:**
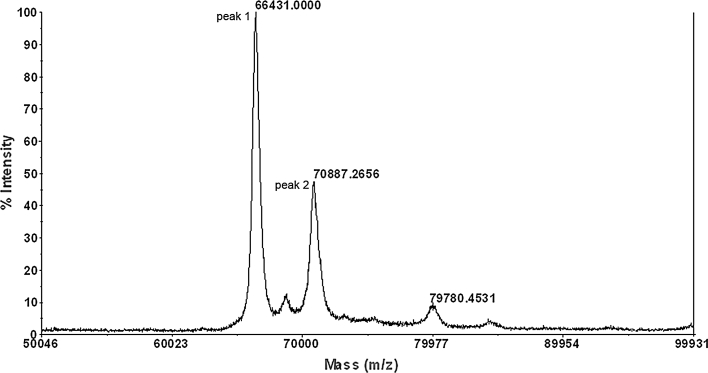
Molecular weight determination of HSA/PTH (1-34) by MALDI-TOF mass spectrometry. Peak 1 represents the BSA internal standard, peak 2 represents HSA/PTH (1-34)

To further confirm that the *i* fragment consists of intact HSA/PTH (1-34), the purified *i* fragment was analyzed by MALDI-TOF mass spectrometry. As shown in Fig. 6. The *i* fragment showed a molecular weight of 70,887.2656 Da, which is very close to the theoretical molecular mass deduced from its amino acid sequence (70,887.49 Da).

### Effect of different protease-deficient strains on proteolysis by shake flask cultivation

**Fig. 7:**
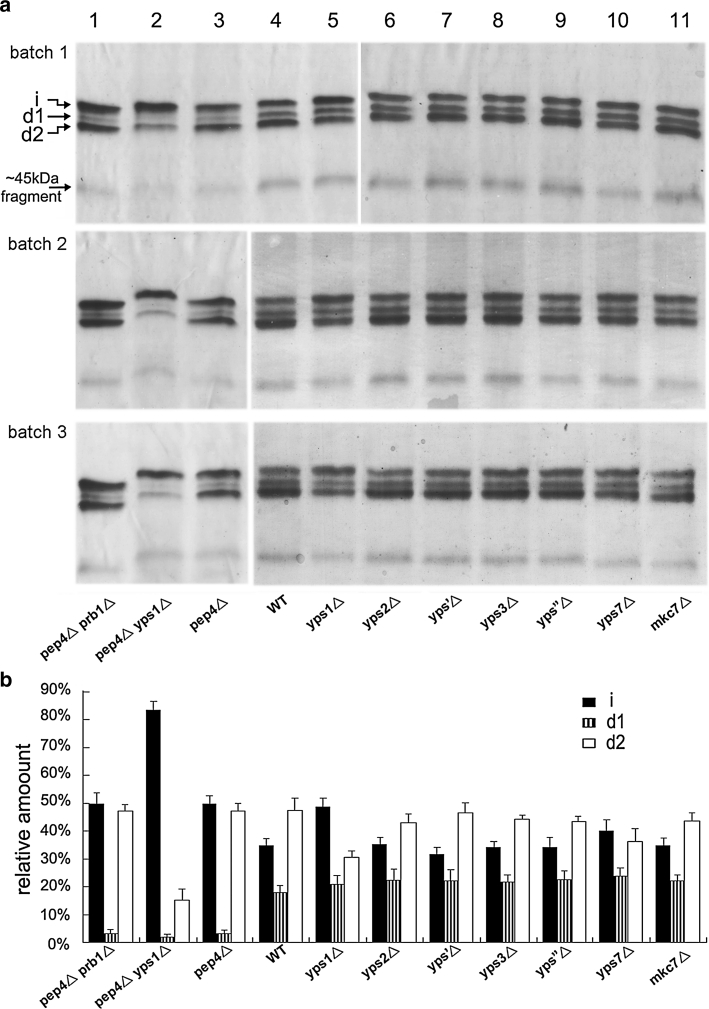
PAGE analysis of recombinant HSA/PTH (1-34) secreted through different protease-deficient strains. **a** Native PAGE gels of three independent batches of cultivation. For each strain, cells were induced in BMMY medium for 72 h and the equivalent of 20 μl of clarified supernatant was loaded for all samples. **b** The relative abundance of intact protein (*i*) and cleaved protein *(d1* and *d2)* was determined by scanning with Quantity One Software version 4.5.0 (Bio-Rad), values were indicated as mean ± S.D, from three independent batches of cultivation. Calculation of ~45 kDa fragment was not included since densitometry of ~45 kDa fragment at low optical density was unreliable

To explore the effect of each yapsin member in proteolysis of HSA/PTH (1-34), seven single-yapsin-deficient GS115 mutants were constructed and tested. For each strain, a clone containing one copy of integrated HSA/PTH (1-34) gene was selected for the comparable cultivation (verified by quantitative real time-PCR, data not shown). Three batches of culture supernatants were obtained for densitometry and statistical analysis after 72 h induction. As shown in Fig. 7 (lane 4–11), among the seven single-yapsin-deficient strains, the proportion of intact HSA/PTH (1-34) was increased significantly only in *yps1*△ disruptant, in which the degradation fragment d2 was considerably diminished but not completely disappeared, while no visualized change was observed for *d1* fragment. For the other six single-yapsin-deficient strains, the band patterns of each disruptant were quite similar with wild-type strain when visualized from PAGE gels. For the seven single-yapsin-deficient strains, culture supernatants after 24 h induction were also analyzed, and the results were similar to 72 h induction, that is the *yps1*△ disruptant displayed a visible reduction of fragment *d2* while the effects of the other six single-yapsin-deficient strains were not significant (see Fig. S1). These observations suggest that yapsin 1 played a role in proteolysis of HSA/PTH (1-34), particularly in generation of fragment *d2*.

Proteinase A and proteinase B are two major vacuolar proteases which also regulate activities of many other vacuolar proteases such as carboxypeptidase Y [11], thus disruptants GS115 *pep4*△ (proteinase A deficient) and GS115 *pep4*△*prb1*△ (proteinases A and B deficient) were also investigated. As shown in Fig. 7, fragment *d1* was diminished significantly in strain GS115 *pep4*△, compared to wild-type strain GS115, but no change was observed for fragment *d2* (Fig. 7, lane 3, 4). A similar expression pattern was found in GS115 *pep4*△*prb1*△ as in GS115 *pep4*△ (Fig. 7, lane 1, 3). These results suggest that proteinase A is involved in the formation of fragment *d1* in a direct or indirect way, while proteinase B is less important. Besides, we found that the *pep4*△ disruptant seemed somewhat beneficial for reduction of ~45 kDa HSA-truncated fragment. This observation was more discernible in another albumin fusion protein HSA/IL1Ra (Fig. S2).

From the above results, we found that *PEP4* disruption benefited the elimination of *d1* fragment and *YPS1* disruption was beneficial for reduction of *d2* fragment. Thus, we further constructed a double disruptant GS115 *pep4*△*yps1*△ and tested its effects for the production of HSA/PTH (1-34) fusion protein. The results showed that disruption of *PEP4* and *YPS1* genes yielded a significant increase in the proportion of intact HSA/PTH (1-34) by inhibition of degradation fragments *d1* and *d2*, as compared to the wild-type host and single disruption of either *PEP4* or *YPS1* (Fig. 7, lane 2), with 80 % of the secreted product remaining intact in *pep4*△*yps1*△ (Table 2). The percentage of intact HSA/PTH (1-34) produced in other strains was about 47 % for both *pep4*△ and *pep4*△*prb1*△, 42 % for *yps1*△, and 30 % for GS115 wild-type host (Table 2).

**Table 2:** Yield of intact protein in wild type and *PEP4*/*YPS1* disruptant strains

Strain	Yield of intact protein (%)^a^
WT	30 ± 2
*pep4*△	47 ± 3
*pep4*△*prb1*△	47 ± 3
*yps1*△	42 ± 3
*pep4*△*yps1*△	80 ± 3

^a^Data are mean ± S.D., acquired from three independent batches of cultivation. For each batch, supernatants of each strain were obtained after 3d-induction at 30 °C and subjected to native PAGE analysis. Yields were quantified by scanning with Quantity One Software version 4.5.0 (Bio-Rad)

### Fed-batch fermentation of GS115 *pep4*△*yps1*△ mutant

**Fig. 8:**
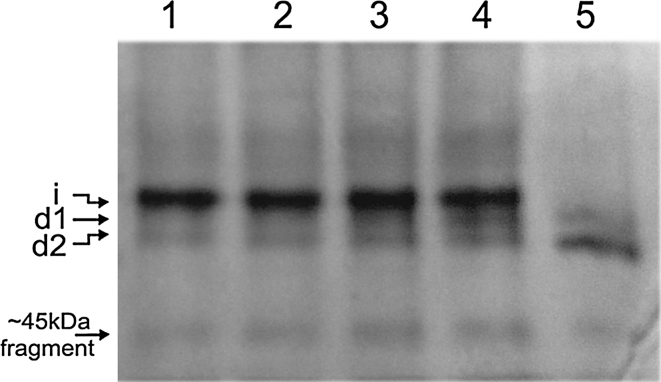
Fed-batch fermentation comparison of GS115 *pep4*△*yps1*△ and wild-type GS115 by native PAGE analysis. *Lane 1–4* supernatants of GS115 *pep4*△*yps1*△ after 21, 25, 29, 33 h methanol induction; *lane 5* supernatants of GS115 after 16 h methanol induction

A bioreactor culture was used to verify the shake flasks results under more controlled conditions. A fed-batch mode was applied and a cellular yield of ~200 g/L wet cells was achieved before methanol induction. Figure 8, for the *pep4*△*yps1*△ mutant, most secreted protein remained intact during the 33 h-fermentation, although degradation products gradually increased after 25 h. However, for the wild-type strain, the intact HSA/PTH (1-34) was almost undetectable as early as after 16 h (and also after 30 h; Fig. S4). This indicates that the *pep4*△*yps1*△ mutant is advantageous over the wild-type by reducing the extent of proteolysis.

## Discussion

The purpose of this study was to construct and develop an effective recombinant expression system in *P. pastoris* for high level production of human PTH (1-34) with HSA as a carrier protein in order to ultimately extend the biological half-life of native PTH (1-34). The fact that severe degradation occurred during HSA/PTH (1-34) production not only resulted in a low yield of intact HSA/PTH (1-34), but also made it more difficult for downstream purification of intact recombinant protein. The proteolytic degradation is partly due to the high level of endogenous protease activity in the host cell, which is elicited by methanol induction causing stress on the cells [21]. Also, extracellular protease levels of *P. pastoris* tend to increase over time [21] and this seems to match our initial observation that the degradation of HSA/PTH (1-34) was dominant at later stage of cultivation.

**Fig. 9:**
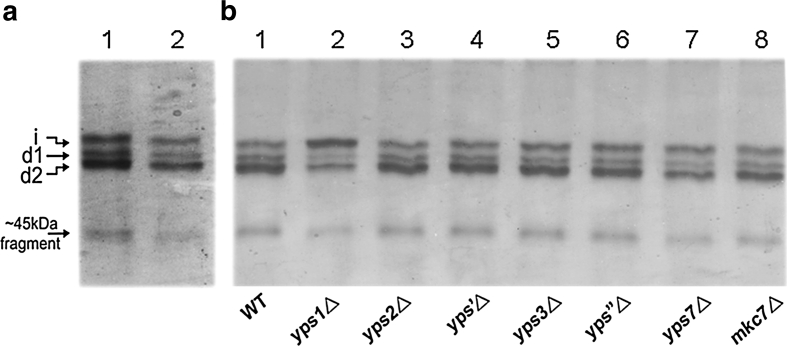
**a** Band patterns comparison between 10 and 20 μl of supernatants from GS115 strain by native PAGE analysis. **b** Band patterns analysis of the seven single-yapsin-disruptants by loading 10 μl of supernatants

In this research, our first attempt was to analyze the effect of each yapsin member on HSA/PTH (1-34) protein production by disruption of the individual genes, and the function of proteinases A and B were evaluated as well (Table 1). Although not sensitive enough to distinguish subtle differences of band patterns, PAGE analyses is a convenient visualized method for comparably evaluating the effects of these suspicious proteases on intact HSA/PTH (1-34) production in this study. As we found that the band patterns of the seven single-yapsin disruptants were quite similar to wild-type GS115 except for *YPS1*△ disruptant, in order to make the densitometry of PAGE bands as accurate as possible, we compared the band patterns between 10 and 20 μl of supernatants from GS115 strain to make sure that interference with density saturation was excluded (Fig. 9a). Besides, supernatants of these single-yapsin-disruptants from one batch were also loaded in 10 μl, and the result was similar as loaded in 20 μl (Fig. 9b).

An important finding in this study is that the *pep4*△ disruptant can efficiently eliminate the prominent cleavage in PTH (17-34) portion (for degradation fragment *d1*). Meanwhile, the proportion of intact HSA/PTH (1-34) was visibly improved by *yps1*△ disruptant, in which the prominent cleavage in PTH (1-17) sequence (for degradation fragment *d2*) was considerably diminished, as compared with wild-type strain. Previous work reported that yapsin activity was almost undetectable in response to a *yps1*△ disruptant, suggesting that yapsin 1 might represent the major yapsin activity in *P. pastoris* [24]. This may partially explain why we did not observe the same positive effect in the other six single-yapsin-deficient strains as in *yps1*△ disruptant. Since the *yps1*△ disruptant alone is not sufficient enough for total elimination of fragment d2, it is possible that all the yapsins synergistically participate in the degradation while activities of yapsin members vary widely. Also, all yapsins recognize basic residues and therefore the exact substrate specificity of the various yapsins and their involvement in the degradation of a particular protein may overlap [18, 19]. Cho et al. [5] also pointed out that though the *yps1*△ disruptant was advantageous over the wild-type strain, the effective prevention of the cleavage of secretory hPTH (1-84) in *S. cerevisiae* was only observed in multiple mutants containing at least the simultaneous disruption of *YPS1*, *YPS2* and *YPS3* genes. Thus, comprehensive analyses of various *YPS* disruption combinations in *P. pastoris* are necessary in further studies to evaluate whether there is an optical combination of multiple-yapsin disruptions for proteolytic inhibition. And also other yapsin-sensitive proteins should also be applied as recombinant protein models.

Yao et al. [26] have reported a minor reduction of the ~45 kDa fragment when they expressed an albumin fusion protein HSA/AX15 (R13 K) by a *yps1*-disrupted *P. pastoris* strain, thus they speculated the existence of other *YPS* homologs in *P. pastoris*. However, in our study, none of the single-yapsin-deficient strains showed significant reduction of the ~45 kDa HSA-truncated fragment. This might partially be caused by the unreliable densitometry analysis (relative to background) of ~45 kDa fragment at low optical density. Thus, the densitometric calculations of the ~45 kDa fragment was excluded. But it is still possible that all the yapsins synergistically participate in the degradation. It is interesting for us to find that the *pep4*△ disruptant seemed somewhat beneficial for reduction of ~45 kDa HSA-truncated fragment (Fig. S2), suggesting the contribution of proteinases A to the formation of ~45 kDa HSA-truncated fragment in *P. pastoris.*

Interestingly, Vad et al. [23] reported in their previous study that no increase in the absolute amount of intact PTH (1-84) was found using strain SMD1163 (GS115 *pep4*△*prb1*△), whereas addition of EDTA to the medium could obtain higher hormone yield. Although the apparent differences between Vad’s observations and ours seem unexplained, we could still speculate that PTH is sensitive to multiple proteases.

Finally, as mentioned above, *pep4*△ disruptant and *yps1*△ disruptant showed the most obvious effects on degradation reduction of HSA/PTH (1-34) fusion protein among the nine selected proteases in the first-round evaluation, thus a double disruptant (*pep4*△*yps1*△) was constructed as the preferred attempt for multiple disruption. The *pep4*△*yps1*△disruptant turned out to be a more effective host for minimizing proteolysis of HSA/PTH (1-34) and for its improved production by *P. pastoris*, as compared to wild-type or single disruption of either *PEP4* or *YPS1*.

We have also tested the *pep4*△*yps1*△ disruptant in fermentation mode. Although degradation products gradually increased after 25 h in *pep4*△*yps1*△ disruptant, the result indicates superiority of this double disruptant over wild-type strain. Of course, to minimize the loss of intact HSA/PTH (1-34), it is also necessary to optimize the fermentation process of *pep4*△*yps1*△ disruptant in further studies.

In the future, to develop a more efficient recombinant protein production system, further investigations are needed for genetic manipulation of *P. pastoris* as well as the optimization of the cultivation conditions. Besides, with the help of genomic sequence project of *P. pastoris*, not only the attempt of multiple-yapsin disruptions but also systematic and comprehensive analyses of a wider range of distinct proteases would be facilitated.

## Electronic supplementary material

Below is the link to the electronic supplementary material.

Supplementary material 1 (DOC 706 kb)
