# Supplementary material for: Disruption of YPS1 and PEP4 genes reduces proteolytic degradation of secreted HSA/PTH in Pichia pastoris GS115
Source: J Ind Microbiol Biotechnol. 2013 Mar 26;40(6):589–99. doi: 10.1007/s10295-013-1264-8 (PMC3656227; doi:10.1007/s10295-013-1264-8)
Supplement: Supplementary file 1 — Supplementary material 1 (DOC 706 kb) [file 10295_2013_1264_MOESM1_ESM.doc]

**Supplementary Materials and Methods**

**1. Construction of the HSA/PTH (1-34) expression vector**

The synthesized PTH (1-34) open reading frame DNA was cloned into plasmid pUC57 and then amplified by PCR using primers PF and PR. cDNA for full-length HSA was amplified from pGEM-T-HSA plasmid (constructed in our laboratory, see reference ) with primers HF and HR. The PCR fragments of PTH (1-34) and HSA were digested with *Bam*H I /*Eco*R I and *Xho* I /*Bam*H I, respectively. The above two digested fragments were then mixed together with *Xho* I/*EcoR* I-cut plasmid fragment pPIC9 and treated with T4 ligase overnight to give the recombinant expression vector pPIC9-HSA/PTH (1-34), in which the HSA/PTH (1-34) expression cassette was joined to the prepro signal of alpha-mating factor. Primers used for construction of HSA/PTH (1-34) expression vector are shown in Table S1.

**2. Production of polyclonal antibodies against PTH and HSA**

The antigen HSA was purchased from sigma. The haptens PTH (1-17) and PTH (17-34) were chemically synthesized and conjugated to BSA to generate BSA-PTH (1-17) and BSA-PTH (17-34) suitable for antibody production.

Two male New Zealand rabbits were immunized subcutaneously on the lower back with HSA in complete Freund’s adjuvant. Another two New Zealand rabbits were immunized with BSA-PTH (1-17) in the same way. And so did the five guinea pigs immunized with BSA-PTH (17-34). After 21 days of the first injection, the animals were injected subcutaneously with the respective immunogen in incomplete Freund’s adjuvant and at 15-day intervals over a period of 4 weeks. Blood samples were taken 6~8 days after each injection and the titer of the respective antisera was determined by indirect ELISA. The antiseras were purified by Protein A chromatography (GE Healthcare) on an ÄKTA purifier system (GE Healthcare).

**Supplementary Table**

**Table S1**. Primers used in this study

| Primer | Sequence (5'-3')^a^ | Amplicon Size |
| --- | --- | --- |
| **Primers for construction of pPIC9-HSA/PTH (1-34)** | | |
| PF | TACGGGATCCTCTGTTTCTG | 122 |
| PR | GCCGGAATTCTTAGAAGTTGTGAAC |  |
| HF | GCCTCGAGAAAAGAGATGCACACAAGAGTGAGG | 1785 |
| HR | CGGATCCACCACCACCTAAGCCTAAGGC |  |
| **Primers for construction of pPICZαB-*YPS*△** | | |
| *YPS1*_NF | AAGCCTGCAGAGCTCCATTGCGCCAACCCC | 257 |
| *YPS1*_NR | CGGCGTCGACAATCTGGCTGAGCGGAAAGTTTGA |  |
| *YPS1*_CF | CCCAGATCTCACATTTCGCGCCTGCCTTCCT | 318 |
| *YPS1*_CR | AAACTGCAGGGGTGAGCCTGTCTGGCCCT |  |
| *YPS2*_NF | AAGCCTGCAG ACGCAACATGCATTGCACAGCC | 231 |
| *YPS2*_NR | CGGCGTCGACTGACTCTTGGTCTAGTTGTTGCCGT |  |
| *YPS2*_CF | CCCAGATCTAGTTCAGATGGAGCTGCCCCGT | 268 |
| *YPS2*_CR | AAACTGCAGCTGAAAAGGTGGTGATCTGCGAAGT |  |
| *YPS3*_NF | AAGCCTGCAGGGCAACATATTGCAGCATTGTGGCA | 307 |
| *YPS3*_NR | CGGCGTCGACAGAAGAAGAGGAGGCCGGCTT |  |
| *YPS3*_CF | CCCAGATCTAGCCACGTTGAAACCATTTACAT | 246 |
| *YPS3*_CR | AAACTGCAGTGCTAGATGTGGCCGCAACCG |  |
| *YPS7*_NF | AAGCCTGCAGAGGCTCGCATAACCCCTCGA | 327 |
| *YPS7*_NR | CGGCGTCGACCAGGGAAGACGAAACATTTGAGCCA |  |
| *YPS7*_CF | CCCAGATCTCTTCTTCAGGAACTCAAACCGAGCA | 327 |
| *YPS7*_CR | AAACTGCAGTCCTGAGGACGATGAGTTCGATCC |  |
| *MKC7*_NF | AAGCCTGCAGAGCAGCATGGCAGCTAACACTGT | 340 |
| *MKC7*_NR | CGGCGTCGACGCCAGCTGGATCACAAACATTCGGT |  |
| *MKC7*_CF | CCCAGATCTAGTTGGATTCTTACTCCGGGTCACT | 321 |
| *MKC7*_CR | AAACTGCAGGGGCGTAGTCAGTTATGTGCTTCA |  |
| *YPS'*_NF | AAGCCTGCAGGGGTACCCTTGGCCTTCTGCG | 303 |
| *YPS'*_NR | CGGCGTCGACGGCACGAGGGTGGTGAAGAATGG |  |
| *YPS'*_CF | CCCAGATCTTGGCTCTGCTTCTGGTACTTCTTCA | 278 |
| *YPS'*_CR | AAACTGCAGACCACCACAGGCAACCTGCAC |  |
| *YPS''*_NF | AAGCCTGCAGACCCTACGCAAACTATAGGGCATGA | 271 |
| *YPS''*_NR | CGGCGTCGACTGATGAAACGTTGTGATCGGTGTGA |  |
| *YPS''*_CF | CCCAGATCTTCCGTACAATGCTTCCGGTGGGA | 268 |
| *YPS''*_CR | AAACTGCAGTGAAGTTTTGCGGCTACCAGCA |  |
| **primers for detection of *yps*-deficient strain** | | |
| *yps1* negative_F | CTCCTTCCACCGCCACGGC | 467 |
| *yps1* negative_R | ACCACTTGAGGCGTCCTTGGA |  |
| *yps2* negative_F | TCAGTCGGTGTACGCAGCGC | 239 |
| *yps2* negative_R | AATTGCTCGCTGACCTCAAGGTC |  |
| *yps3* negative_F | CGGTGACACCACAGCCGAGT | 345 |
| *yps3* negative_R | TGCATTCCTGGTGTGCCCGC |  |
| *yps7* negative_F | CCCCAATGAGTGGAACAACACGGT | 450 |
| *yps7* negative_R | ACGTTCGAAGCGGGAGGAATGC |  |
| *mkc7* negative_F | TCGCCTATGCCATCCATCCTGGT | 499 |
| *mkc7* negative_R | CGTCGAGACGTAGTCGGTTCGC |  |
| *yps'* negative_F | TCCGCATCGGCGACTTTGCC | 296 |
| *yps'* negative_R | TGGCCCGTCTATCTTGGCCCT |  |
| *yps''* negative_F | TGGGCCCACCCAGGGAAAGG | 399 |
| *yps''* negative_R | GCCCCTTGATCAAACCCCACAGC |  |
| *yps1* positive_F | ATGGAGGAGTTGAGGACC | 833 |
| *yps2* positive_F | ACAAGTGCAGTGCTTGTGAC | 644 |
| *yps3* positive_F | CAAAGGGCTTCAAGGACACTG | 579 |
| *yps7* positive_F | TCGTGGCTTAGCCAACCTGA | 598 |
| *mkc7* positive_F | TAATAAAAACGCAGGGCTGTG | 506 |
| *yps'* positive_F | CTATTGCGGCAACCAGCTGTA | 572 |
| *yps''* positive_F | ACTGTGAGCGGGTCCGCCTA | 480 |
| 3’AOX _R | GCAAATGGCATTCTGACATCC |  |

^a^ the underlined sequences in the primers are restriction enzyme sites for subsequent subcloning.

**Supplementary Figures**


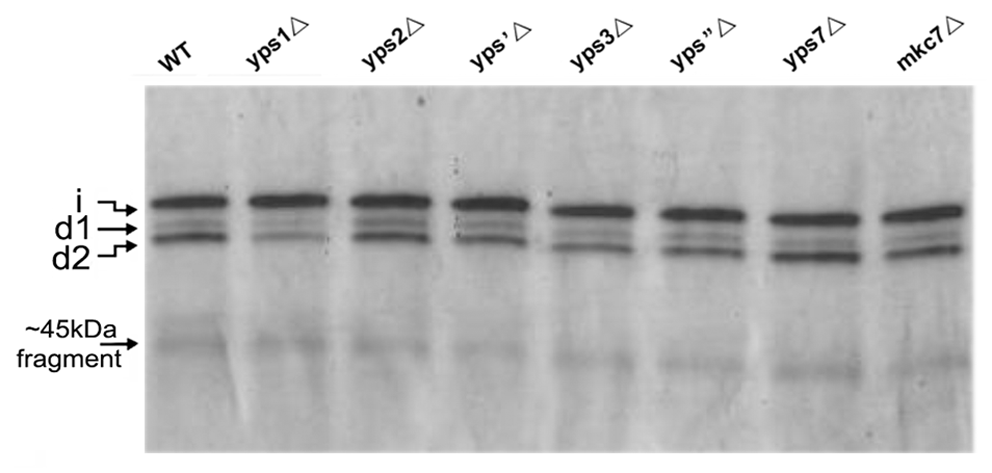


**Fig. S1** Native PAGE analysis of HSA/PTH (1-34) secreted through seven single-yapsin-deficient strains after 24h methanol induction. Because the concentration of HSA/PTH (1-34) in 24h-induced supernatant was too low to form clear bands by Coomassie blue-staining, supernatants from all the strains were concentrated as follows: 1 ml of clarified supernatant was lyophilized (MODULYOD- freeze dryer, Thermo) and then re-dissolved in 150μl water. 20μl of the re-dissolved supernatant was loaded for all samples


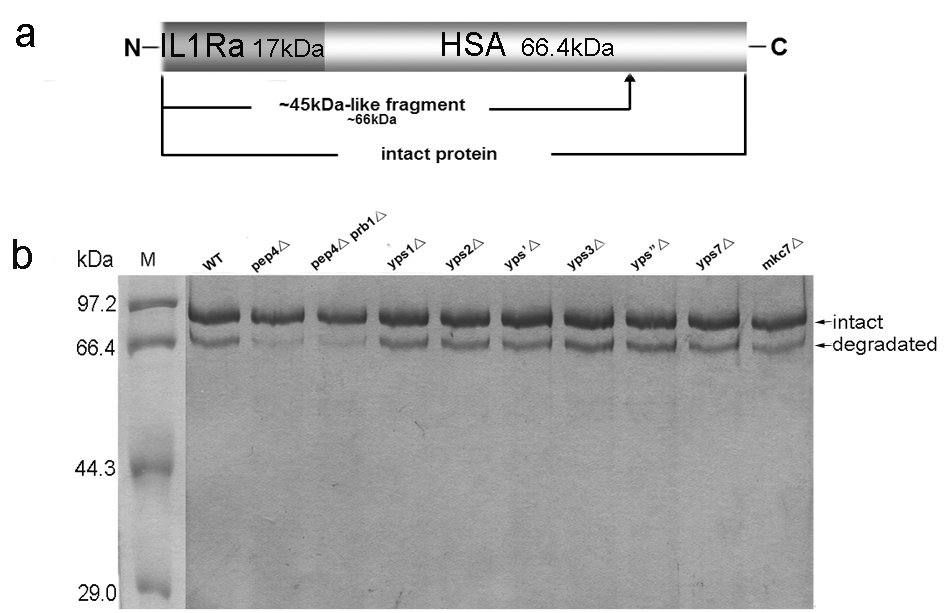


**Fig. S2** (a) Schematic representation of cleavage sites in fusion protein HSA/IL1Ra, the intact and the degradation bands were identified by N-terminal amino acid sequence analysis and MALDI-TOF MS. (b) reducing SDS-PAGE analysis of HSA/IL1Ra fusion protein secreted through different protease-deficient strains. The cultivation condition was the same as HSA/PTH (1-34), and the equivalent of 20μl of clarified supernatant was loaded for all samples


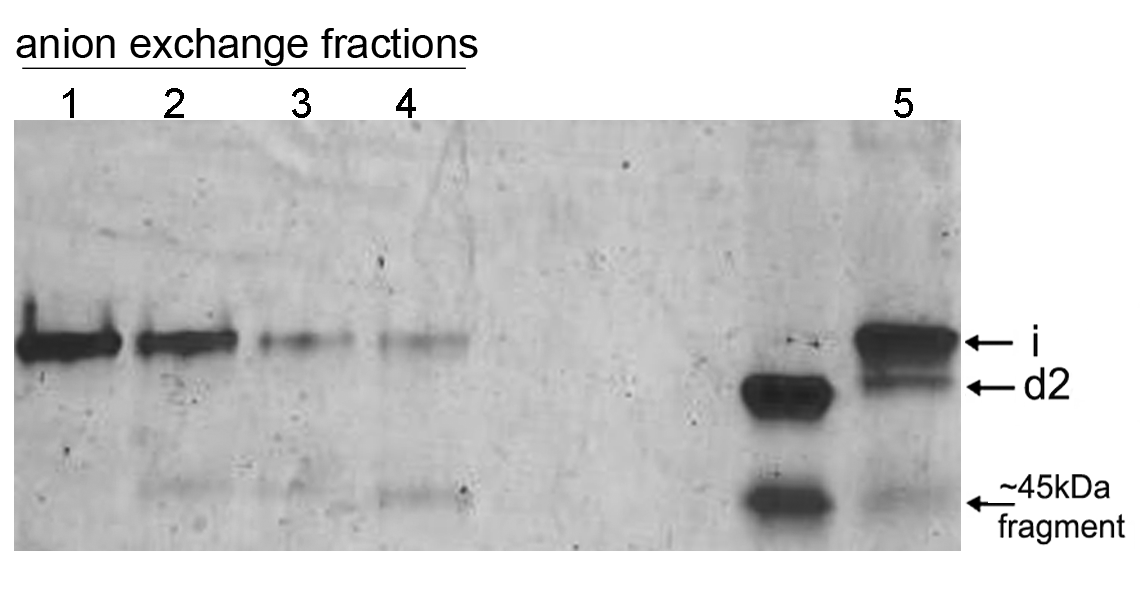


**Fig. S3** Native PAGE analysis of the final chromatography-purified *i* fragment. The supernatant was first applied to a Phenyl Sepharose FF column in the initial capture step. The eluate containing *i* fragment was then further purified on a Q Sepharose XL column. Lane 1~4: Q Sepharose XL anion exchange fractions 1~4; Lane 5: supernatants of GS115 *pep4*△*yps1*△. Fraction 1 was then used for MALDI-TOF mass spectrometry


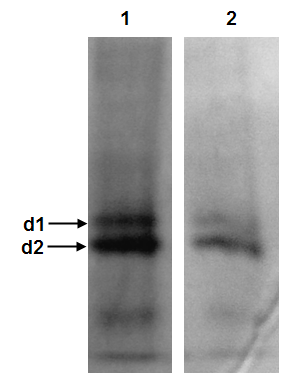


**Fig. S4** Lane 1: fed-batch supernatants of GS115 at 30 h; Lane 2: fed-batch supernatants of GS115 at 16 h

**REFERENCE**

1. Chen J, Sun HY, Yang Y, Wang XF, Chen SQ (2008) Construction, expression and characterization of recombinant fusion protein HSA-PTH (1-34) in Pichia pastoris. Zhejiang Da Xue Xue Bao Yi Xue Ban 37 (2):126-133
